# Case report: Confusing lung signs – is the source of the disease in the lungs or intestines?

**DOI:** 10.3389/fmed.2023.1187208

**Published:** 2023-10-12

**Authors:** Xiying Zhao, Jiahao Mo, Beiping Zhang, Haiyan Zhang

**Affiliations:** ^1^Department of Gastroenterology, The Second Affiliated Hospital of Guangzhou University of Traditional Chinese Medicine, Guangzhou, China; ^2^The Second Clinical Medical College of Guangzhou University of Traditional Chinese Medicine, Guangzhou, China

**Keywords:** Crohn’s disease, granulomatous inflammation, case report, abnormal lung manifestations, lung

## Abstract

At the time of the spread of the COVID-19 epidemic, blurred lung signs suggested by imaging examination are particularly common. Novel coronavirus infection is mainly caused by respiratory symptoms. In the early stage of imaging examination, multiple small patchy shadows or ground glass shadows and invasive shadows of both lungs are dominant. While the pulmonary involvement in Crohn’s disease (CD) is rare and not widely reported. For CD patients, the pulmonary manifestations do not belong to its routine symptoms. The lung involvement of CD patients is difficult to attract clinicians’ attention. If CD patients have vague lung manifestations but have no response to routine treatment, they should consider the respiratory diseases related to CD. We describe a rare case of granulomatous inflammation associated with Crohn’s disease. The patient do not respond to conventional treatment. The final treatment plan was CD immunomodulatory therapy (oral corticosteroids and azathioprine). After treatment, a review of the lung CT showed focal fibrosis and significant improvement in the lung lesions. It suggests that CD related respiratory diseases should be considered when CD patients have abnormal lung manifestations that do not respond to conventional treatment.

## Introduction

At the time of the spread of the COVID-19 epidemic, the imaging examination of the patients showed that the lung blurred symptoms were particularly common (1). While pulmonary involvement in Crohn’s disease (CD) is clinically rare. We present a rare case of CD involving the lungs. It should be reminded that in our clinical work, when patients with CD encounter abnormal lung manifestations that are ineffective in conventional treatment, we need to pay high attention to the correlation between CD and lung disease. For these patients with respiratory symptoms and a history of CD, despite the pulmonary imaging symptoms, the source of the disease may be intestinal disease. If so, treatment should be considered for intestinal diseases rather than traditional anti-infection treatment of lung (2). This point deserves the attention of clinicians.

## Case description

**Figure 1:**
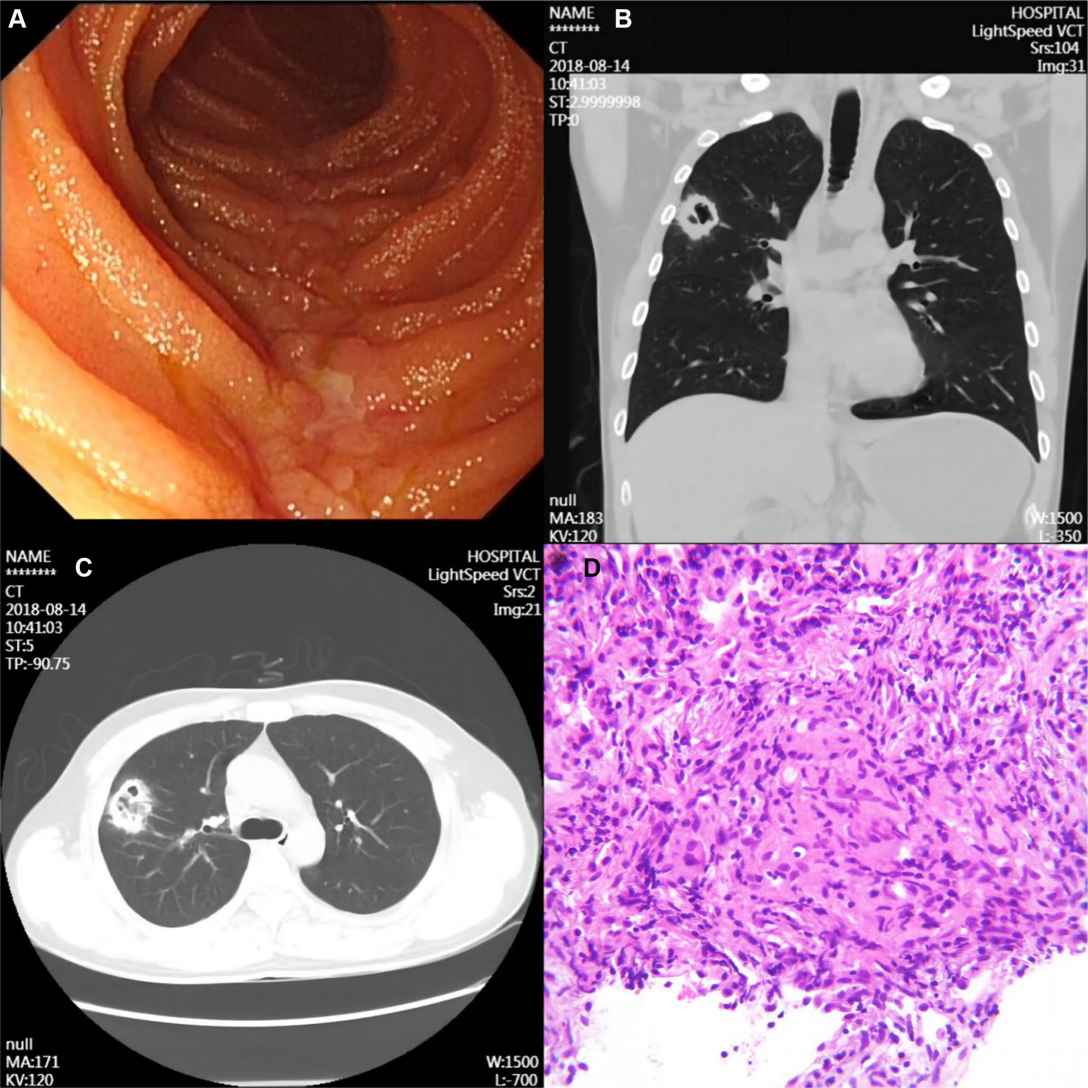
Imaging and pathological findings of the patient. **(A)** Typical longitudinal ulcer in transanal small bowel microscopy. **(B)** Representative CT scan of the chest showing a mass-like abnormal density shadow in the anterior segment of the upper lobe of the right lung. **(C)** Chest CT scan of a mass measuring approximately 3.6 cm × 3.0 cm in the anterior segment of the upper lobe of the right lung with unrefined margins. **(D)** Lung puncture biopsy specimen shows epithelioid histiocytes, fibroblast proliferation, a small amount of lymphocytic infiltration, and multinucleated giant cells and non-necrotic epithelioid granuloma.

A 36-year-old male patient was admitted to the hospital with abdominal pain for 1 year and perianal exudate for 3 months. Crohn’s disease (CD) was suspected. The patient had not taken any medication for CD. Enteroscopy showed that there were several longitudinal depressions with a length of about 4 cm-10 cm in the ileum about 2 m and 1.8 m away from the ileocecal valve and at the end of the ileum, surrounded by paving stones (Figure 1A). The detailed enteroscopy results were as follows: the single balloon enteroscopy was performed along the lumen to the place about 2 mm from the ileum to the Ileocecal valve. When the microscope was withdrawn, the ileum was about 2 m and 1.8 m from the Ileocecal valve and the end of the ileum was covered with white moss, with many longitudinal sunken ulcers about 4 cm-10 cm long, and the surrounding area was like paving stones. Irregular ulcer in ring 1/2 cavity could be seen about 80 cm from the ileum to the Ileocecal valve, covered with yellow white fur. The remaining ileal mucosa showed scattered irregular concave ulcers with a length of approximately 3 mm to 6 mm, covered with white fur. The mucosa between the ulcers was normal. Four biopsy specimens were taken from the ileal ulcer site and sent for pathology. The Ileocecal valve could see three irregular ulcers of about 4 mm-7 mm in shape, covered with white fur. Irregular concave ulcers with a diameter of about 2 mm to 4 mm could be seen scattered in the Ascending colon, Transverse colon, Sigmoid colon and rectum. The mucosa around the white coating was congestive and edematous, and the mucosa between the ulcers was normal. The patient’s previous medical history: In December 2017, the patient underwent surgical treatment for a sudden intestinal perforation. The surgical method was “laparoscopic exploration+open small intestine partial resection+intestinal adhesiolysis.” During the surgery, multiple ulcers were found on the mesenteric side of the distal ileum, with some ulcers exceeding 10 cm in length. The surgery was successful. Postoperative pathology showed: fissure ulcer was seen at the lesion, transmural inflammation was seen in the whole intestinal wall, pyloric gland metaplasia was seen in the mucosal layer, multiple foci of histiocytes were gathered, and epithelioid Granuloma was seen, a large number of Lymphatic vessel were dilated in the submucosa, mucosal muscle structure was disordered, multiple foci of Lymphoid hyperplasia were seen in the whole intestinal wall, with the formation of lymph follicles, fibrous tissue hyperplasia in the serosa layer, accompanied by the formation of inflammatory granulation tissue, and a large number of inflammatory exudates were seen on the surface of the serosa layer. The exudates were mainly neutrophils. Based on the intraoperative findings, it is preliminarily considered Crohn’s disease. Postoperative medication history: From December 2017 to August 2018, there was no medication treatment before the follow-up examination. The diagnosis of CD was confirmed by comprehensive evaluation and pathological examination. The patient has no history of smoking. Computed tomography (CT) of the lungs revealed nodular and mass-like abnormal density shadows (Figure 1B), the largest was located in the anterior segment of the upper right lobe and measured 3.6 × 3.0 cm, with unrefined margins (Figure 1C).

**Figure 2:**
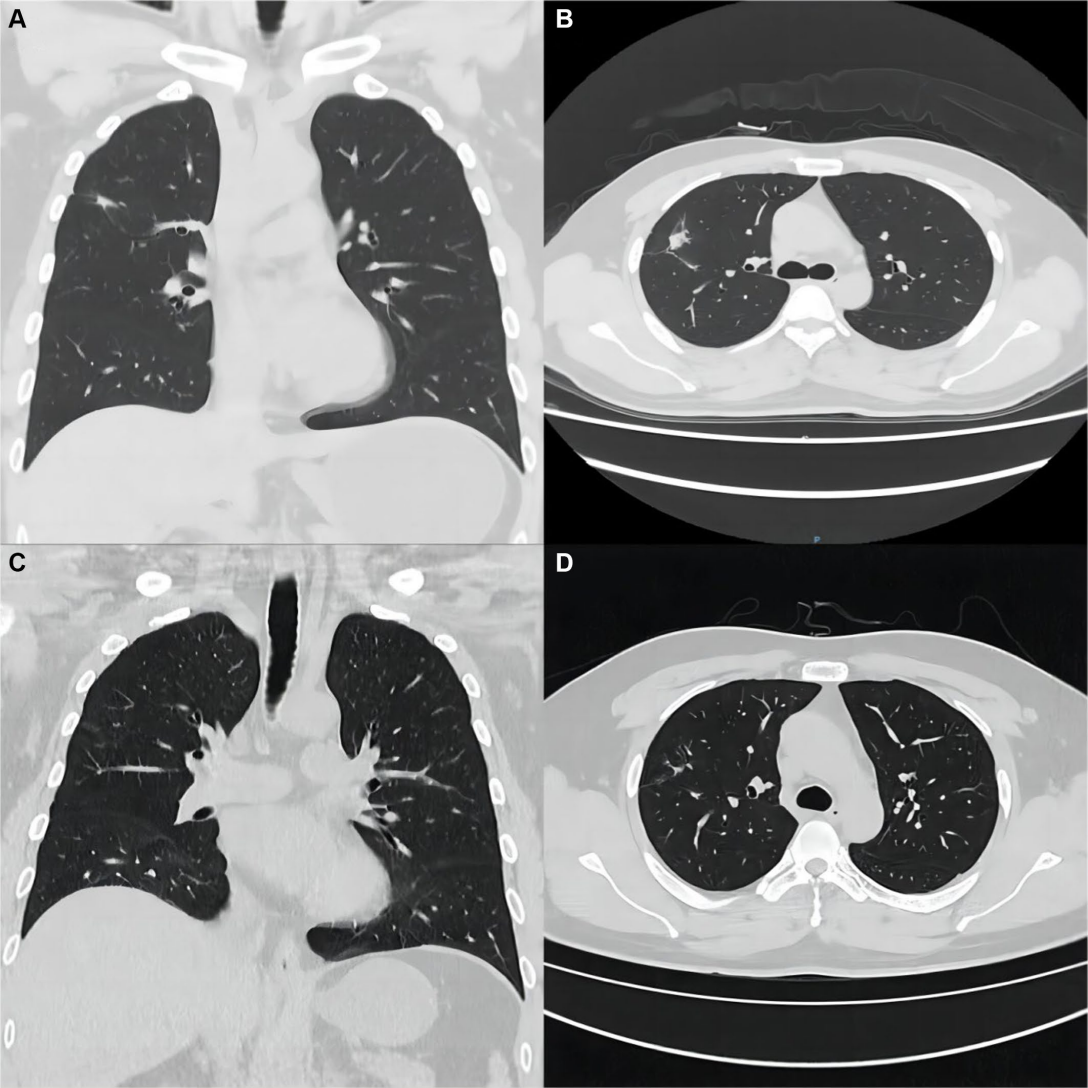
Imaging and pathological findings of the patient at the time of return visit. **(A,B)** After 6 months of treatment, a repeat chest CT showed significant improvement of the original lung lesion. **(C,D)** CT scan of the chest at follow-up until May 2022, with only a few fibrous lesions remaining from the original lung lesion.

Blood and sputum pathogenic tests and an aspiration biopsy of the right lower lung lesion were done during hospitalization. Tests for bacteria, fungi, viruses, mycoplasma titer, and Legionella antigen were negative. Combining the clinical and special staining findings, we diagnosed the lesion of the lung as granulomatous inflammation (Figure 1D). Immunohistochemical results were as follows: TTF-1 (epithelial +), CK7 (epithelial +), CD68 (histiocyte +), ALK (−), and SMA (few +). Special staining results were as follows: antacid (−), PAS (−), and PASM (−). The special staining results are shown in Supplementary Figure S1. Bacterial, fungal, mycobacterial, and cytological analyses showed the absence of cancer cells and infection. These findings were consistent with the diagnosis of CD-associated granulomatous pneumonia. Based on the above judgment, the patient started immunomodulatory therapy with oral corticosteroids and azathioprine. CT scans 6 months after discharge showed significant improvement of the pulmonary lesions (Figures 2A,B), indicating that these were associated with CD. During the next 2+ years of follow-up, infliximab maintenance therapy was changed in 2021 for CD; follow-up has been stable to date. The patient’s pulmonary lesions remained asymptomatic, and a lung CT in May 2022 suggested focal fibrosis (Figures 2C,D).

## Discussion

Pulmonary involvement in CD patients is rare in clinical practice and its pathogenesis is unclear (3). Structural similarities between the intestine and bronchi and their common origin in the primitive foregut may contribute to the bronchial inflammatory changes in patients with inflammatory bowel disease (4). For CD patients, the pulmonary manifestations do not belong to its routine symptoms. The lung involvement of CD patients is difficult to attract clinicians’ attention. First, such extraintestinal lesions are rare. Secondly, in anatomical position, due to the distance between the lung and the intestine, it is not easy to associate the nodular and lumpy abnormal density shadows of the lung CT with intestinal lesions. If CD patients have vague lung manifestations but have no response to routine treatment, they should consider the respiratory diseases related to CD. Previous literature has suggested that patients with CD have acute respiratory distress syndrome and pulmonary fibrosis as a complication of surgery, with chest radiographs showing bilateral pulmonary infiltrates (commonly in the upper middle lobe) accompanied by bilateral pleural effusions (5). A study by Raghava also has also found airway involvement in patients with CD (6). However, apart from these two reports, there are few clinical reports related to lung involvement in CD within the previous 5 years. Therefore, we think it is very important to report this one case.

In this case, we present a case of granulomatous pneumonia due to CD lung involvement. The final treatment plan was CD immunomodulatory therapy (oral corticosteroids and azathioprine). After treatment, a review of the lung CT showed focal fibrosis and significant improvement in the lung lesions. Two lessons can be learned from this case: firstly, in patients with abnormal density shadows on CT of the lungs, we should be concerned about the patient’s other existing diseases in addition to the lungs themselves; secondly, when a patient with CD develops pulmonary abnormalities during treatment, we should distinguish between primary lung disease and CD-related respiratory disease, especially after conventional treatment has failed. The present case clearly demonstrates the benign outcome of CD lung involvement leading to granulomatous pneumonia.

At the time of the spread of the COVID-19 epidemic, blurred lung signs suggested by imaging examination are particularly common. In the early stage of imaging examination, multiple small patchy shadows or ground glass shadows and invasive shadows of both lungs are dominant. Therefore, in clinical practice, attention should be paid to those patients with respiratory symptoms who have a history of inflammatory bowel disease. Despite the appearance of imaging pulmonary signs, the source of the disease is actually intestinal disease. The treatment should also aim at intestinal diseases rather than conventional lung anti-infection treatment. This case deserves the attention of clinicians.

## Data availability statement

The original contributions presented in the study are included in the article/Supplementary material, further inquiries can be directed to the corresponding author.

## Ethics statement

Written informed consent was obtained from the individual(s) for the publication of any potentially identifiable images or data included in this article.

## Author contributions

XZ and JM conceived and designed the report. JM and BZ collected the data. XZ and JM reviewed the literature and wrote the manuscript. XZ and HZ revised and edited the manuscript. All authors contributed to the article and approved the submitted version.

## References

[1] Pan X Zhu H Du J Hu G Han B Jia Y. MS-DCANet: a novel segmentation network for multi-modality COVID-19 medical images. J Multidiscip Healthc. (2023) 16:2023–43. doi: 10.2147/JMDH.S417068 37489133 PMC10363353

[2] Lin L Pengwei L Xiaoping N He C. Interstitial lung disease as an Extraintestinal manifestation of Crohn's disease in the time of COVID-19: a rare case report and review of the literature. J Inflamm Res. (2022) 15:5733–7. doi: 10.2147/JIR.S380879 36238767 PMC9553307

[3] Kraft SC Earle RH Roesler M Esterly JR. Unexplained bronchopulmonary disease with inflammatory bowel disease. Arch Intern Med. (1976) 136:454–9. doi: 10.1001/archinte.1976.03630040056012, PMID: 1267553

[4] van Lierop PP Samsom JN Escher JC Nieuwenhuis EE. Role of the innate immune system in the pathogenesis of inflammatory bowel disease. J Pediatr Gastroenterol Nutr. (2009) 48:142–51. doi: 10.1097/MPG.0b013e3181821964 19179875

[5] Sofia C Adiletta V Iovene B Sgalla G Richeldi L. Acute respiratory distress syndrome and lung fibrosis complicating surgery in a patient with Crohn's disease. Inflamm Bowel Dis. (2022) 28:e31–2. doi: 10.1093/ibd/izab255, PMID: 34730805

[6] Raghava Rao G Vishal S Kashinath MS Dhooria S Sehgal IS Prasad KT. Tracheobronchial Crohn's disease: case report and systematic review of the literature. Inflamm Bowel Dis. (2022) 28:e33–5. doi: 10.1093/ibd/izab258, PMID: 34730812

