# Supplementary figures and images for: Case report: Confusing lung signs – is the source of the disease in the lungs or intestines?
Source: Front Med (Lausanne). 2023 Oct 12;10:1187208. doi: 10.3389/fmed.2023.1187208 (PMC10602771; doi:10.3389/fmed.2023.1187208)

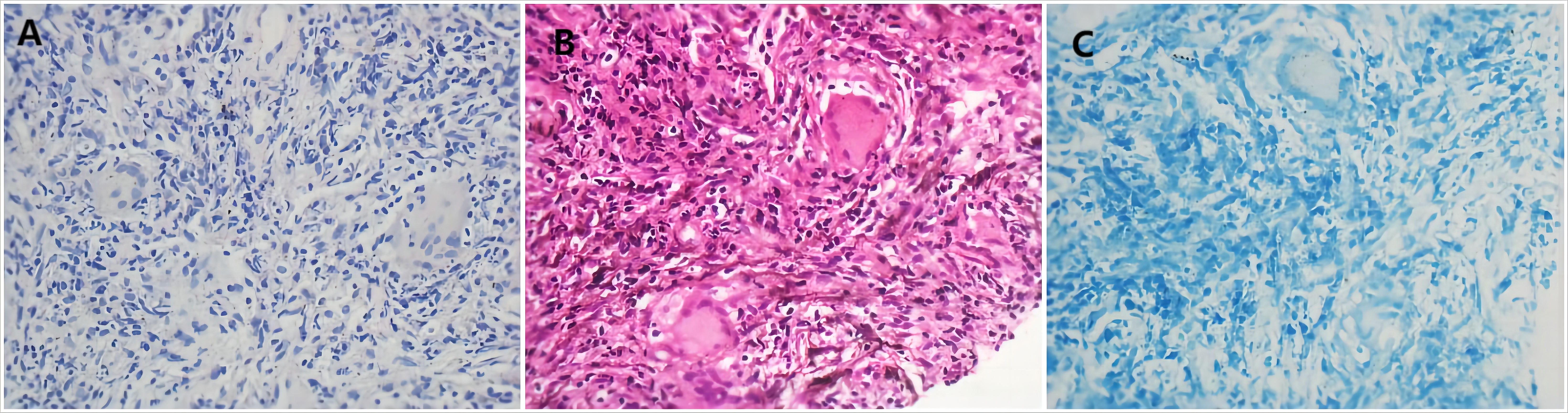

Supplement: Supplementary file 1 [file Image_1.jpeg]
